# Phytochemical Analysis of *Tephrosia vogelii* across East Africa Reveals Three Chemotypes that Influence Its Use as a Pesticidal Plant

**DOI:** 10.3390/plants8120597

**Published:** 2019-12-12

**Authors:** Angela G. Mkindi, Yolice Tembo, Ernest R. Mbega, Beth Medvecky, Amy Kendal-Smith, Iain W. Farrell, Patrick A. Ndakidemi, Steven R. Belmain, Philip C. Stevenson

**Affiliations:** 1 Department of Sustainable Agriculture, Biodiversity and Ecosystems Management, Centre for Research, Agricultural Advancement, Teaching Excellence and Sustainability (CREATES), The Nelson Mandela African Institution of Science and Technology, P.O. Box 447 Arusha, Tanzaniaernest.mbega@nm-aist.ac.tz (E.R.M.); patrick.ndakidemi@nm-aist.ac.tz (P.A.N.); 2 Bunda College, Lilongwe University of Agriculture and Natural Resources-Malawi, P.O. Box 219 Lilongwe, Malawi; ytembo@bunda.luanar.mw; 3 Innovations in Development, Education and The Mathematical Sciences (IDEMS) International, 15 Warwick Road, Reading, RG2 7AX, UK; 4 Royal Botanic Gardens, Kew, Richmond, Surrey TW9 3DS, UK; bs16a3s@leeds.ac.uk (A.K.-S.); i.farrell@kew.org (I.W.F.);; 5 Faculty of Biological Sciences, University of Leeds, Leeds LS2 9JT, UK; 6 Natural Resources Institute, University of Greenwich, Central Avenue, Chatham Maritime, Kent ME4 4TB, UK

**Keywords:** spatial-temporal variation, chemotype 3, deguelin, rotenoids, botanical insecticides

## Abstract

*Tephrosia vogelii* is a plant species chemically characterized by the presence of entomotoxic rotenoids and used widely across Africa as a botanical pesticide. Phytochemical analysis was conducted to establish the presence and abundance of the bioactive principles in this species across three countries in East Africa: Tanzania, Kenya, and Malawi. Analysis of methanolic extracts of foliar parts of *T. vogelii* revealed the occurrence of two distinct chemotypes that were separated by the presence of rotenoids in one, and flavanones and flavones that are not bioactive against insects on the other. Specifically, chemotype 1 contained deguelin as the major rotenoid along with tephrosin, and rotenone as a minor component, while these compounds were absent from chemotype 2, which contained previously reported flavanones and flavones including obovatin-3-*O*-methylether. Chemotype 3 contained a combination of the chemical profiles of both chemotype 1 and 2 suggesting a chemical hybrid. Plant samples identified as chemotype 1 showed chemical consistency across seasons and altitudes, except in the wet season where a significant difference was observed for samples in Tanzania. Since farmers are unable to determine the chemical content of material available care must be taken in promoting this species for pest management without first establishing efficacy. While phytochemical analysis serves as an important tool for quality control of pesticidal plants, where analytical facilities are not available simple bioassays could be developed to enable extension staff and farmers to determine the efficacy of their plants and ensure only effective materials are adopted.

## 1. Introduction

*Tephrosia vogelii* Hook. f. (*Leguminosae*) is a plant species reported to be used widely for its medicinal, insecticidal, and soil enrichment potential in tropical Africa [1,2,3,4,5,6]. Specifically, research on *T. vogelii* reported medicinal properties such as anti-cancer activity [7,8,9] and efficacy as an ectoparasite treatment for domestic animals including poultry [10,11,12,13,14]. A number of studies have sought to validate the reported use of *T. vogelii* as a botanical insecticide under laboratory and field conditions and have reported its effectiveness for crop protection and reduced impacts on beneficial ecosystem services [15,16,17]. Likewise, *Tephrosia* is reported to have high biomass and is therefore important as a soil amendment and is compatible with food crops when intercropped in addition to its nitrogen fixing property [18,19]. Hence, using *T. vogelii* for small scale farmers may support reduced industrial fertilizer and synthetic pesticides application all of which bear cost and safety implications.

Deguelin was reported to be the major active compound in *T. vogelii* occurring in all plant parts along with the minor components of tephrosin and rotenone [20,21,22,23]. However, a previous study reported that some *T. vogelii* did not contain rotenoids, and was less effective as an insecticide [15,22]. This highlighted the need to ensure that effective chemotypes of pesticidal plants were available when promoting their use to farmers to ensure effective control of pests. However, this is challenging in the absence of suitable local facilities to undertake such quality control and establish variation when pesticidal plants are harvested, processed, and used locally [24]. Natural variation in the chemistry of bioactive components in pesticidal plants is reported [25] and can have consequences for use and ultimately trust in pesticidal plants as an alternative to synthetic inputs by farmers.

Variability in the chemistry of *T*. *vogelii* could lead to farmers unknowingly using ineffective material and influence negatively the wider adoption and commercialization of botanical insecticides. Small scale farming communities, who are the main beneficiaries of *T. vogelii,* identify the plant using locally acquired knowledge through morphological features. However, *T. vogelii* chemotypes may not be identified and distinguished morphologically [22]. Likewise, traditional use does not have the capacity to determine effectiveness prior to use. Phytochemical analysis is an essential tool for the selection of elite provenances of plant materials [26] and should be used for the identification of effective *T. vogelii* provenances prior to propagation. Here we collected samples from local farmers who had *Tephrosia* growing in their fields and were using it for some non-food purpose. We sought to understand the key applications of *Tephrosia* through a survey of farmers who used the plants. From the collected samples, we evaluated the presence and concentration of deguelin in *T. vogelii* leaf materials across 91 locations in three East African countries to establish the extent of chemotype variation and identify elite materials for propagation of improved seed material of *T. vogelii.* Variation of *Tephrosia* chemotypes with flower colors, seasons, rainfall, and altitudes were also assessed to establish whether these traits could be used as markers of effectiveness or for the presence of active chemicals in the plant. Further recommendations are also presented to help farmers more easily identify bioactive plants for improved efficacy.

## 2. Results and Discussion

### 2.1. Status of Use of *Tephrosia vogelii* by Small Scale Farmers

**Figure 1:**
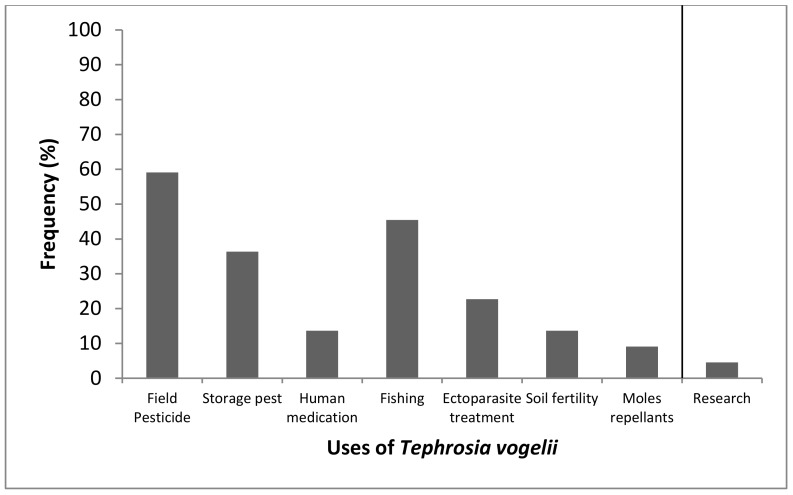
Ethno botanical uses of *T. vogelii* among local small-scale farmers from the six Tanzanian regions. Data are frequencies of responses on eight key uses of *T. vogelii* from the sample size (*n* = 22).

Eight questions were used to assess the extent of *T. vogelii* use among farmers in locations where samples were collected in Tanzania. All interviewed farmers were aware of *T. vogelii*, commonly known as “Utupa” (Figure 1.). Farmers reported using *T. vogelii* mostly for field pest control (59%), fishing (45%), and storage pest control (36%). Other uses such as, human medication, soil fertility and as mole repellants and for ectoparasites control were also reported. A few farmers (5%) reported awareness of the plant from witnessing institutional researchers collecting the plant for use in research and also planting *Tephrosia* in research institutions such as the Uyole Agricultural Research―Mbeya and Tanzania Coffee Research Institute―Kilimanjaro. The specific research activity was not clearly communicated.

Our results indicate that *T. vogelii* is most widely used for pest control among other uses as shown in the survey results. Farmers’ responses in this study, align with reports about the use of *T. vogelii* in controlling pests in vegetables and in stored products [15] and ectoparasite control in domestic animals [12,24,27]. The wider use of *T. vogelii* for small-scale farmers could be associated with previous projects that promoted integrated pest management using *T. vogelii* and research on soil improvement [28,29].

### 2.2. Phytochemical Analysis of T. vogelii Leaf Samples

Analysis of methanolic extracts of the leaf samples identified 2 chemotypes. Figure S1, shows the LC-MS chromatograms of chemotype 1 characterised by the presence of rotenoids with corresponding peaks between 19 and 20 min. These were determined from a comparison of their spectral data to in house standards [22]. Rotenone was identified from UV (LC-PDA) λmax nm, 301; (MS) *m/z*, 395.4 [M + H]^+^, while tephrosin was identified from UV (LC-PDA) λmax nm, 272, 300, 314 sh; (MS) *m/z*, 433.4 [M + Na]^+^ and deguelin from UV (LC-PDA) λmax nm, 270, 301, 319; (MS) *m/z*, 395.4 [M + H]^+^. Chemotype 2 with peaks between 18 and 21 min were determined to contain obovatin 3-*O*-methylether as the major component from an in house standard and had UV (LC-PDA) λmax nm, 270, 295, 348; (MS) *m/z*, 337.4 [M + H]^+^. Other similar components having ions with *m/z* = 337 and 367 corresponding to flavones and flavanones are reported earlier including *Z*-tephrostachin [22].

We also identified a third chemotype (Figure S1). Chemotype 3 was a chemical hybrid of chemotypes 1 and 2 showing the presence of both the rotenoids and the flavanones and flavones reported in 1 and 2 respectively in equivalent quantities. A further finding recorded plants as chemotype 1 but indicated trace quantities of flavones and flavanones from chemotype 2, suggesting that a variety of potential chemical variants may exist in natural and propagated materials. The analyses were undertaken on a single leaflet so were not a consequence of sample mixing of chemotypes 1 and 2. Furthermore individual leaves from the same plant were chemically very similar.

The presence of chemotypes 1 and 2 corresponded with findings [22] in which chemotypes 1 and 2 were first reported. However, here we provide a comprehensive regional assessment of their occurrence to determine what potential impacts the occurrence of chemotype 2 might have on the application and uptake of this species for pest control and other uses. Chemotype 3 is a previously unreported chemotype in this species which we report here. Studies have shown that the production of new flavonoids such as those found in *T. vogelii* could be influenced by environmental factors whereby compounds changed after exposure to conditions such as carbohydrates and light [20]. However, chemical variation in these varieties is most likely genetic since different chemotypes were first reported from the same location and in the same soil in adjacent fields [22]. There would therefore be value in analysing for further chemotypes and determining if the hybrids produce lower quantities of both compound groups.

### 2.3. Frequency of T. vogelii Chemotypes.

Plant material was collected from specific locations in three countries (Tanzania, Kenya, and Malawi) where *T. vogelii* is used and their geographical references are presented in Table S1. Approximately, 7% of samples were identified as chemotype 3, while 20% were chemotype 2. Most samples (74%) were chemotype 1. A higher proportion of chemotype 1 was also reported by [15] in Malawi from the analysis of 12 samples. Table 1, illustrates the proportions of chemotypes by countries. The abundance in plant materials with chemotype 1 coincided with efficacy studies of *T. vogelii* on medicinal [10,13,21,30] and insecticidal properties of rotenoids [15,16,17,31] which revealed that rotenoids were the compounds most frequently found in *T. vogelii* sampled and are responsible for the plants’ biological activity.

### 2.4. Spatial Distribution of Plants Samples Chemotypes

**Figure 2:**
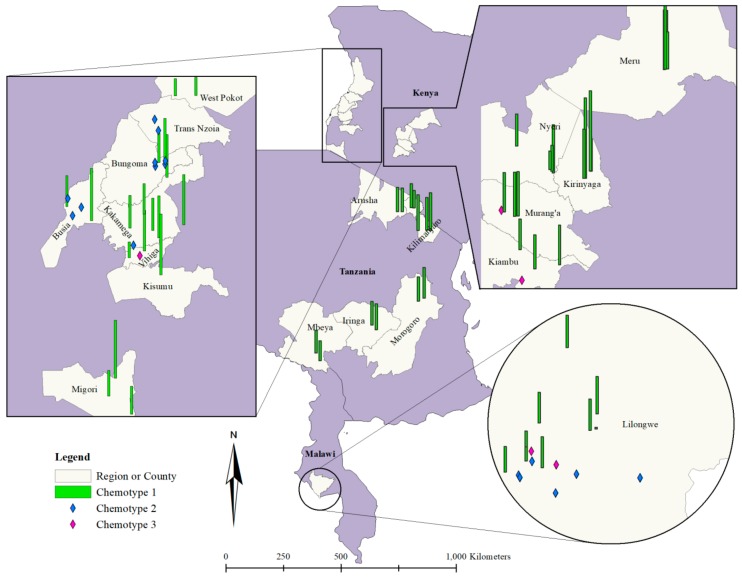
Spatial variation of *T. vogelii* chemotypes in Tanzania, Kenya, and Malawi, indicating presence of Chemotypes 1, 2, and 3. Green bars depict the presence of deguelin while blue marks indicate the presence of chemotype 2. The purple marks indicates the presence of chemotype 3, a chemical hybrid of chemotype 1 and 2.

In this study, deguelin, the most abundant pesticidal rotenoid in *T. vogelii*, was used as an indicator compound and its concentration in the plant was assessed across the study zone. The chemical composition of *T. vogelii* was presented with reference to location across the three countries (Figure 2). Samples collected from Malawi and Kenya contained chemotypes 1, 2, and 3 and were located in Lilongwe and in 12 Kenyan counties respectively, while only chemotype 1 was recorded from 14 locations across five regions in Tanzania. The results, from this study present the potential for understanding the diversity of pesticidal plant chemotypes across a whole region. Local efficacy testing of pesticidal activity in *T. vogelii* using a simple assay would potentially be conducted across various locations where the plants grow to ensure reliable efficacy results for local farmers using the plant. 

### 2.5. Spatial Temporal Variation of Chemotype 1 in T. vogelii

Linear regression analysis was performed to test the variation of deguelin content in *T. vogelii* based on altitude. The linear regression for Malawi (r^2^ = 0.178, F = 3.9, df = 18, *p* = 0.064), Kenya (r^2^ = 0.03, F = 1.74, df = 56, *p* = 0.193), dry season in Tanzania (r^2^ = 0.008, F = 0,096, df = 12, *p* = 0.762), and wet season in Tanzania (r^2^ = 0.122, F = 1.665, df = 12, *p* = 0.221) showed no significant relationship between changing altitude and the concentrations of deguelin. Further analysis of data from Tanzania revealed no significant correlation between rainfall recorded in the wet (r^2^ = 0.005, F = 0.016, df = 3, *p* = 0.9), and dry seasons (r^2^ = 0.72, F = 7.725, df = 56, *p* = 0.069). The results concur with findings reported by [15] although these earlier data were of just a few samples.

Analysis of variance on the samples collected over two seasons in Tanzania showed that there was no significant variation in the deguelin concentration with locations in the dry season (ANOVA F = 0.272, df = 8, *p* = 0.916). In the wet season, however, a significant variation (ANOVA F = 7.092, df = 8, *p* = 0.008) was observed (Table 2) where the highest and lowest levels of deguelin were observed in samples collected from Same and Mbeya districts respectively. Seasonal variation of deguelin was also reported by [15,32] where higher concentrations occurred in the wet season compared with the dry season.

### 2.6. Association between T.vogelii Flower Color and Chemotypes

**Figure 3:**
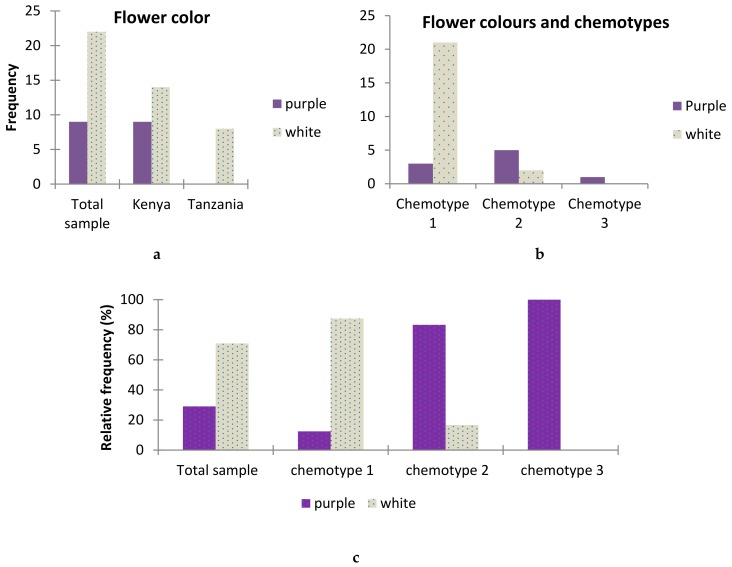
The figure above shows (**a**) a descriptive statistics results showing the presence of purple and white flowers in Tanzania and Kenya; (**b**) numerical distribution of *T. vogelii* flower color with the chemotype of the plant; and (**c**) the frequency in percentage (%) of the occurrence of flower color with chemotype.

One simple morphological feature for identification of *T. vogelii* and potentially distinguishing chemotypes is flower color, with colors typically white or purple. In this study, plants that had flowers at the time of sample collection were recorded along with leaf samples for chemical analysis. Generally, a high percent of plants with white colored flowers were recorded compared to those with purple flower. A higher percent of occurrence of white flowers was associated with the presence of chemotype 1. Chemotype 3 was associated with only purple color while a lower percent of white color was associated with chemotype 2 (Figure 3). Regression analysis showed a strong correlation (r^2^ = 0.43, F = 22.02, df = 29, *p* = 0.0001) between chemotype and flower color where chemotype 1 was related to white and chemotype 2 to purple color. In contrast to earlier findings [22] who found no correlation. Flower colors could be used as initial tool for identification of chemotypes. However, the fact that some purple flowers (with smaller percent) were also associated with chemotype 1, the decision to use a plant for pest control purposes should be guided with simple assays for evidence of effective chemotypes. Small scale farmers could adopt simpler tests of plant materials against storage pests such as cowpea weevils (*Callosobruchus maculatus*) as already done by Belmain et al. [15] although this was under laboratory setting.

### 2.7. Summary of Indicators for Chemotype Identification

Small-scale farmers require hands-on information to enable them to decide on suitable *T. vogelii* materials for pest control. From this study, proposed and tested indicators would be used. Table 3 below highlights what farmers would need to consider while harvesting and using the materials.

## 3. Materials and Methods

### 3.1. Analysis of Tephrosia Vogelii Leaf Samples

#### 3.1.1. Samples Collection

Plant leaf samples were collected from farmers’ fields. Farmers identified the specific *T. vogelii* plants that were used for controlling crop pests and diseases and for medical uses. In Tanzania, leaf samples of *T. vogelii* were collected over two seasons, the wet season and dry season in 14 sites located across five regions: Arusha, Kilimanjaro, Morogoro, Mbeya, and Iringa. The five regions were identified after revising *T. vogelii* collections preserved in the National Herbarium in Tanzania to identify possible areas where the plants could be growing. Samples were collected in March and September 2018 during the wet and dry season respectively. In each region, two sites were identified where samples were collected depending on the availability of the plant at that time.

Herbarium samples were collected and assigned voucher numbers, processed and stored in the National herbarium. Identified plant in each point of sample collection was used for the two seasons to justify analysis of variation with seasons. Rainfall data for the particular months of sample collection were obtained from the Tanzania Meteorological Agency (TMA)―Tanzania. In Malawi, samples were collected in the Lilongwe area on farmers’ fields. Likewise in Kenya collections were made on farmers’ fields in Kisumu, Homa Bay, Migori, Siaya counties, the western region Kakamega, Busia, Bungoma, Mumias, and Central Kenyan counties.

A total of 28 samples were collected in Tanzania that included 14 samples for each of the dry and wet seasons. In Malawi, 20 samples were collected from Lilongwe area between May and November 2018, while in Kenya, a total of 57 samples were collected between February and April 2019. Collected samples were dried under the shed, packed into plastic zip bags and stored in dark and dry conditions under ambient temperature before being processed and analyzed.

#### 3.1.2. Survey of Farmers Awareness on the Use of *T. vogelii*

Twenty two farmers from six Tanzanian regions were interviewed in the survey to determine the uses of *T. vogelii* in the household. In order to identify *T. vogelii* uses with reference to the type of sample collected, only farmers who owned the plant or neighbors to the famer owning the plants were interviewed. The selection of farmers therefore did not follow specific social survey protocols for sample sizes selection.

#### 3.1.3. Sample Analysis

Dried *T. vogelii* samples were powdered using an electric grinder (SALTER, Model No EK2311ROFB distributed by UP Global Sourcing, Victoria Street, Manchester, OL9 0DD, UK Made in China). *Tephrosia* powder (50 mg/mL) was extracted in methanol. Each extract was left to stand for 24 h at room temperature before chemical analysis. Plant leaf extracts were transferred to Eppendorf tubes and centrifuged for 20 min at 5000 rpm. ***S***upernatant (300 uL) was transferred into HPLC vials for analysis. Extracts were analyzed by liquid chromatography (LC)-Electrospray Ionization Mass Spectroscopy (ESIMS) and UV spectroscopy using Thermo Fisher Velos Pro LC-MS. Samples (5 μL) were injected directly on to a Phenomenex Luna C18 (2) column (150 Å~3 mm i.d., 3 μm particle size) at 400 μL min^−1^ and eluted using a linear gradient of 90:0:10 (t = 0 min) to 0:90:10 (t = 20–25 min), returning to 90:0:10 (t = 27–30 min). Solvents were water: methanol: 1% formic acid in acetonitrile, respectively. The column was maintained at 30 °C. Compounds were detected on a Thermo Fisher Velos Pro Dual-Pressure Linear Ion Trap Mass Spectrometer. Samples were scanned, using FTMS, from *m/z* 200–600 corresponding to the range of molecular ions expected in samples of *T. vogelii.* UV peak area were quantified against a calibration curve of an authentic in-house standard [22]. The resulting peak areas of deguelin were measured at a wavelength of 300 nm and arranged in an excel file for statistical analysis. 

### 3.2. Presentation of Data and Sampling Points

Graphical presentation of chemotype and variation in amounts of chemotype 1 in *T. vogelii* was performed using ARC GIS, ARCMAP version 10.3.

### 3.3. Statistical Analysis

Analysis of Variance and descriptive statistics, proportion analysis and regression analysis were performed using XLSTAT version 2019.2.2.59614 (Addinsoft (2019). XLSTAT statistical and data analysis solution. Boston, MA, USA. https://www.xlstat.com).

## 4. Conclusions

This study has demonstrated the chemical variation in *T. vogelii*, across a variety of location types in three countries of East Africa and revealed considerable variation in chemistry influencing the bioactivity of plants materials. The study has also highlighted key uses of the plant, hence indicating its importance to farmers’ livelihoods. Correlation of key factors with the effectiveness of the plant materials are also discussed along with the identification of options that farmers would consider when selecting elite materials. From this study we realize the potential of a region wide study that provide an expanded perspective of plant chemistry for a wider community use and uptake. To mitigate the variations under local conditions, simple and locally tailored assays, where farmers could test plant materials against storage pests would provide a rapid assessment tool of plants efficacy. However, further research on possible propagation strategies that ensure the availability and use of elite materials as well as investigation of more indicators for chemotype identification is required.

## Figures and Tables

**Table 1:** Summary distribution of chemotype within the study area.

Variables	No. of Observations	No. of Missing Values	No. of Categories	Mode	Mode Frequency	Categories	Frequency Per Category	Rel. Frequency Per Category (%)	Proportion Per Category
Overall	91	0	3	Chemotype 1	67	Chemotype 1	67	74	1
						Chemotype 2	18	20	0
						Chemotype 3	6	7	0
Kenya	57	0	3	Chemotype 1	44	Chemotype 1	44	77	1
						Chemotype 2	10	18	0
						Chemotype 3	3	5	0
Malawi	20	0	3	Chemotype 1	9	Chemotype 1	9	45	0
						Chemotype 2	8	40	0
						Chemotype 3	3	15	0
Tanzania	14	0	1	Chemotype 1	14	Chemotype 1	14	100	1
						Chemotype 2	0	0	0
						Chemotype 3	0	0	0

**Table 2:** Spatial and temporal variation of deguelin in *T. vogelii* from locations in Tanzania. The values presented are means ± SE. **, = significant at *P* ≤ 0.01, ns = not significant. Means followed by the same letter in a column are not significantly different.

Location	Dry Season Deguelin (ppm)	Wet Season Deguelin (ppm)
Same	6841 ± 523 a	8756 ± 197 a
Iringa	5644 ± 1202 a	4879 ± 132 bc
Morogoro	5423 ± 1621 a	6229 ± 207 b
Kilimanjaro	5144 ± 682 a	6377 ± 791 b
Mbeya	5699 ± 314 a	3385 ± 196 c
Arusha	5339 ± 139 a	4803 ± 4 bc
One way ANOVA F statistics	0.27 ns	7.09 **

**Table 3:** Tested and proposed options that farmers would need consider to select effective *T. vogelii* plant material.

Option	Results	Reliability for Chemotypes Identification
**Tested Options**		
Elevation	No correlation	Not reliable
Season	No correlation	Not reliable: Although wet season enhances higher content of bioactive compounds in chemotype 1
Flower Color	Positive correlation	Somewhat reliable: Could be used to decide on the chemotype where white flowers are known to be related with chemotype 1. N.B., a few plants with chemotype 1 had purple flowers.
**Proposed Options**		
Simple assays	Report from Belmain et al., 2012	Reliable: Test assessment of plant (10% leaf powder in small test container with bruchids), could be a rapid, simple and affordable tool. Pesticidal properties of *Tephrosia* are fast acting and chemotype could be determined in 48 h.

## Supplementary Materials

The following are available online at https://www.mdpi.com/2223-7747/8/12/597/s1, Table S1: Location and chemotype data for samples of *T. vogelii* in Tanzania, Kenya, and Malawi, Figure S1: Chromatograms showing: (**a**) Chemotype 1, presence of deguelin; (**b**) Chemotype 2 absence of rotenoids; (**c**) chemotype 3 signifying presence of chemotypes 1 and 2 suggesting a hybrid, and (**d**) presence of Chemotype 1 with minimal quantitative presence of chemotype 2.
