# Supplementary material for: Phytochemical Analysis of Tephrosia vogelii across East Africa Reveals Three Chemotypes that Influence Its Use as a Pesticidal Plant
Source: Plants (Basel). 2019 Dec 12;8(12):597. doi: 10.3390/plants8120597 (PMC6963982; doi:10.3390/plants8120597)
Supplement: Supplementary file 1 [file plants-08-00597-s001.pdf]

Table S1: Location and chemotype data for samples of *T. vogelii* in Tanzania, Kenya and Malawi.

Farmer/Sample ID	Country	Latitude	Longitude	altitude
01-K	Kenya	0.097752	37.81782	1168
02-K	Kenya	0.325643	34.53851	1340
03-K	Kenya	0.979937	35.15177	1892
04-K	Kenya	0.090201	37.80688	1171
05-K	Kenya	-0.526584	37.14551	1663
06-K	Kenya	1.011569	34.81895	1944
07-K	Kenya	0.428555	34.83557	1541
08-K	Kenya	-1.10017	34.50181	1367
09-K	Kenya	0.498218	34.12219	1141
10-K	Kenya	-0.559879	37.3352	1314
11-K	Kenya	-0.750621	36.83641	2208
12-K	Kenya	0.873637	34.87782	1747
13-K	Kenya	-0.95086	34.32468	1274
14-K	Kenya	-0.759193	36.85265	2165
15-K	Kenya	-0.367204	36.92607	1865
16-K	Kenya	0.049257	34.68665	1561
17-K	Kenya	-0.558601	37.32853	1300
18-K	Kenya	0.757884	34.86166	1662
19-K	Kenya	-1.167437	36.95979	1510
20-K	Kenya	0.432866	34.22514	1177
21-K	Kenya	0.437587	34.11315	1184
22-K	Kenya	0.743374	34.78629	1619
23-K	Kenya	0.115114	34.63049	1474
24-K	Kenya	0.36835	34.15812	1156
25-K	Kenya	0.112597	34.62899	1478
26-K	Kenya	0.110225	34.81027	1639
27-K	Kenya	0.211953	34.82631	1530
28-K	Kenya	-0.517841	37.37005	1413
29-K	Kenya	-1.074544	37.1823	1452
30-K	Kenya	1.098055	34.78232	2137
31-K	Kenya	1.014987	34.81005	1997
32-K	Kenya	1.176398	34.93681	1869
33-K	Kenya	-1.098766	37.03414	1504
34-K	Kenya	-1.099615	34.43201	1342
35-K	Kenya	0.257286	34.76586	1475
36-K	Kenya	-0.517195	37.13713	1684
37-K	Kenya	0.06437	34.66981	1548
38-K	Kenya	-0.515672	37.36573	1398
39-K	Kenya	0.093449	37.81905	1155

40-K	Kenya	0.758926	34.87193	1680
41-K	Kenya	-0.78523	36.91462	1928
42-K	Kenya	-0.080452	34.82601	1150
43-K	Kenya	-1.136357	34.60511	1389
44-K	Kenya	0.143038	34.62155	1430
45-K	Kenya	0.091232	37.81236	1164
46-K	Kenya	0.274213	34.59082	1310
47-K	Kenya	-0.506081	37.12583	1706
48-K	Kenya	0.092842	37.82547	1160
49-K	Kenya	-0.985336	36.94538	1625
50-K	Kenya	0.77217	34.81198	1635
51-K	Kenya	-0.965147	34.48188	1393
52-K	Kenya	0.331085	34.3008	1269
53-K	Kenya	0.756035	34.85681	1651
54-K	Kenya	-0.783291	36.93494	1751
55-K	Kenya	1.278913	35.09086	2075
56-K	Kenya	0.784362	34.86308	1685
57-K	Kenya	0.771604	34.78512	1689
01-M	Malawi	-14.19613	33.76749	1182
02-M	Malawi	-14.21196	33.75928	1173
03-M	Malawi	-14.1534	33.78207	1108
04-M	Malawi	-14.18489	33.77386	1131
05-M	Malawi	-14.20842	33.74425	1147
06.M	Malawi	-14.19637	33.77437	1182
07-M	Malawi	-14.19637	33.77437	1146
08-M	Malawi	-14.23192	33.80106	1185
09-M	Malawi	-14.19613	33.76749	1182
10-M	Malawi	-14.16169	33.83935	1124
11-M	Malawi	-14.20348	33.78574	1166
12-M	Malawi	-14.21442	33.76104	1182
13-M	Malawi	-14.16032	33.8459	1123
14-M	Malawi	-14.19999	33.80179	1164
15-M	Malawi	-14.21487	33.8955	1146
16-M	Malawi	-14.2108	33.8244	1173
17-M	Malawi	-14.06896	33.81357	1085
18-M	Malawi	-14.19637	33.77437	1182
19-M	Malawi	-14.21487	33.8955	1146
20-M	Malawi	-14.1431	33.84702	1107
01-TZ	Tanzania	-3.40427	36.79448	1174.3
02-TZ	Tanzania	-3.40427	36.79447	1183.9
03-TZ	Tanzania	-8.0209	35.8564	1883.3
04-TZ	Tanzania	-7.83219	35.84432	1677
05-TZ	Tanzania	-4.137065	37.9056	1399

06-TZ	Tanzania	-3.2511	37.24033	1254.9
07-TZ	Tanzania	-4.135077	37.91367	1181
08-TZ	Tanzania	-3.26394	37.35318	1289.3
09-TZ	Tanzania	-8.920116	33.52334	1788
10-TZ	Tanzania	-9.232094	33.63924	1448
11-TZ	Tanzania	-6.83867	37.74604	764.57
12-TZ	Tanzania	-6.84718	37.69085	899
13-TZ	Tanzania	-4.142703	37.90506	1428
14-TZ	Tanzania	-4.136123	37.91188	1202

Figure S1: Chromatograms showing: (a) Chemotype 1, presence of deguelin; (b) Chemotype 2 absence of rotenoids; (c) chemotype 3 signifying presence of chemotypes 1 and 2 suggesting a hybrid and d Presence of Chemotype 1 with minimal quantitative presence of chemotype 2.

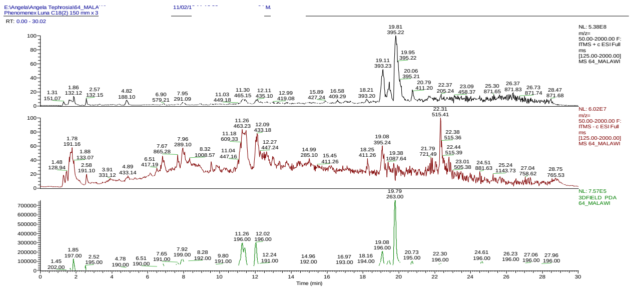

(a)

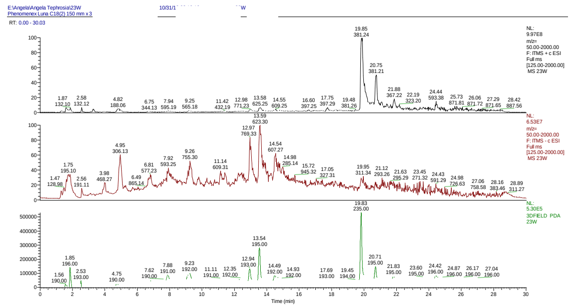

(b)

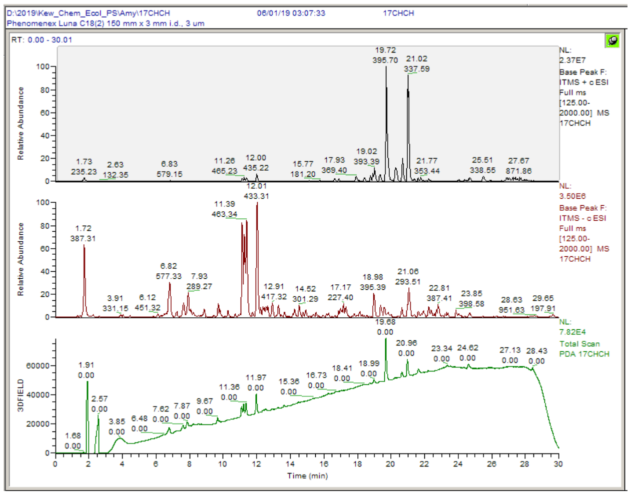

(c)

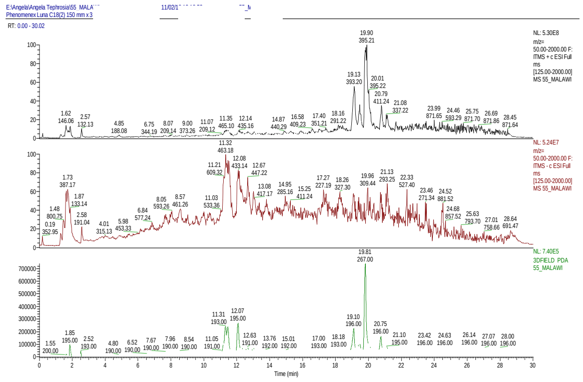

(d)
